# Fracture of the inferior pole of the patella: tension band wiring versus transosseous reattachment

**DOI:** 10.1186/s13018-021-02519-x

**Published:** 2021-06-08

**Authors:** Chih-Hsun Chang, Hao-Chun Chuang, Wei-Ren Su, Fa-Chuan Kuan, Chih-Kai Hong, Kai-Lan Hsu

**Affiliations:** 1 Department of Orthopaedic Surgery, National Cheng Kung University Hospital, College of Medicine, National Cheng Kung University, 138 Sheng-Li Rd., Tainan, Taiwan, Republic of China; 2 Skeleton Materials and Bio-compatibility Core Lab, Research Center of Clinical Medicine, National Cheng Kung University Hospital, College of Medicine, National Cheng Kung University, Tainan, Taiwan, Republic of China; 3 Division of Orthopaedics, Department of Surgery, National Cheng Kung University Hospital Dou Liou Branch, National Cheng Kung University, Yunlin, Taiwan, Republic of China; 4 Department of Biomedical Engineering, National Cheng Kung University, Tainan, Taiwan, Republic of China; 5 Division of Traumatology, National Cheng Kung University Medical Center, Tainan, Taiwan, Republic of China

**Keywords:** Patellar fracture, Inferior pole, Tension band wire, Transosseous reattachment

## Abstract

**Background:**

The optimal surgical technique for the fixation of inferior pole patellar fracture remains controversial. The aims of this study were (1) to compare clinical and radiological outcomes following fixation of inferior pole patellar fracture by using tension band wire (TBW) and transosseous reattachment (TOR) without excision of the bony fragment and (2) to determine the risk factors for postoperative radiological loss of reduction.

**Methods:**

For this retrospective cohort study, consecutive patients with inferior pole patellar fracture between January 2010 and December 2017 were recruited. The patients were grouped according to their fixation method (TBW or TOR), and demographic data, clinical outcomes, and postoperative Insall–Salvati (IS) ratio were analyzed. Then, the patients were grouped according to radiological loss of reduction, the possible risk factors for loss of reduction were identified, and odds ratios were calculated.

**Result:**

This study included 55 patients with inferior pole patellar fracture; 30 patients were treated using TBW and 25 were treated using TOR. Clinical failure occurred in two patients in the TBW group (7%) and three in the TOR group (12%). The rate of radiological loss of reduction was significant higher in the TOR group, whereas removal of implants was significantly more common in the TBW group. Patella baja was noted immediately after surgery in the TOR group, but the IS ratios of the two groups were similar after 3 months. Fracture displacement of more than 30 mm was the only independent risk factor for postoperative radiological loss of reduction.

**Conclusion:**

For treating inferior pole patellar fracture, both TWB and TOR were effective and had a low clinical failure rate. In 60% of patients undergoing TBW fixation, however, additional surgery was required to remove the implants. Patella baja occurred immediately following TOR, but the patellar height was similar to that in the TBW group after 3 months. Surgeons should be aware of the high risk of postoperative radiological loss of reduction, especially when the fracture displacement is more than 30 mm.

## Introduction

Fractures of the inferior pole of the patella are a unique type of patellar fracture in which the patella is extra-articularly avulsed by the patellar tendon. Such fractures account for 5 to 22.4% of all patellar fractures [1, 2] and are usually comminuted [3]. Surgical treatment is recommended for displaced fractures of the inferior pole of the patella to restore the extensor mechanism of the lower extremity. Various techniques have been proposed for treating such comminuted fractures, including tension band wiring (TBW) [4], wiring through cannulated screws [5], separate vertical wiring, augmentation with a rim plate, cerclage wiring or suture [6–8], plate fixation [9–12], partial patellectomy with transosseous reattachment (TOR) [13], and suture anchoring [2–14]. Although excellent outcomes have been reported, most have been obtained from case series; few studies have compared the clinical results of the aforementioned methods.

Among these surgical techniques, TBW and TOR may be the most commonly performed because they are technically easier than the other methods and do not require special implants. TBW is a traditional treatment for transverse patellar fracture. Some surgeons also use TBW in treating inferior pole patellar fractures even when the fragment is small or comminuted, but few results have been reported [4, 5]. Conversely, TOR with partial patellectomy is a treatment for inferior pole patellar fracture, especially when the fragment is too small or comminuted to be fixed. However, the clinical outcomes following TOR with partial patellectomy have varied [15, 16]. Additionally, TOR with partial patellectomy has been associated with patella baja [10–17] and thus may result in unfavorable functional outcomes [10]. To prevent this complication, we preserved all fragments of the patella’s inferior pole to reduce the original patella height and encourage bone-to-bone healing at the fracture site. However, comparisons of the two commonly used surgical techniques—TBW and TOR—through assessment of radiological and clinical outcomes remain limited.

Therefore, the aims of this study were (1) to compare the clinical outcomes—including rate of radiological loss of reduction, nonunion, and clinical failure—between TWB and TOR for fixation of inferior pole patellar fracture, (2) to compare the Insall–Salvati (IS) ratio after TWB and TOR, and (3) to identify the risk factors for radiological loss of reduction after the open reduction and fixation of inferior pole patellar fracture.

## Method

### Participants

This retrospective cohort study was approved by our institution’s institutional review board. Patients were retrospectively recruited from a medical center in southern Taiwan between January 2010 and December 2017. We used the coding system of the Taiwan National Health Insurance, and all medical charts were reviewed. The inclusion criteria were closed fracture of the inferior pole of the patella with a displacement of more than 2 mm and treatment comprising surgical fixation through TBW or TOR. Fracture of the inferior pole of the patella was defined as extra-articular involvement with proximal extension of less than half of the patella’s height on a superficial surface. We excluded patients with skeletal immaturity, with insufficient (< 6 months) follow-up, who underwent a revision procedure, with fracture managed using other surgical techniques, and with additional implants for augmentation.

### Surgical method and postoperative rehabilitation

Operations were performed by several surgeons who employed similar techniques. The operation method was decided by the surgeon in accordance with their preference. The TBW construct comprised two longitudinal 1.6- or 1.8-mm Kirschner wires (K-wires) across the fracture line and one stainless steel anterior tension band. The parallel K-wires were shortened after bilateral bending [18] to prevent them from becoming dislodged. Moreover, TOR was performed using three or four 2.0-mm transosseous tunnels running from the fracture site to the upper pole of the patella. The patellar tendon was stitched with two No. 5 Ethibond sutures by using the Krackow technique, and the threads were passed proximally through the tunnel. The threads were tied to achieve bone contact at the inferior pole fracture site with the knee in full extension. We use the term TOR instead of partial patellectomy because the fragments over the inferior pole were not excised to ensure bone-to-bone healing and preserve the patella’s height. Furthermore, the ruptured retinaculum was repaired using No. 5 Ethibond sutures in both the TBW and TOR groups.

Postoperative rehabilitation programs were similar in the two groups but differed slightly between patients. Partial weight bearing and extension with knee-brace immobilization were allowed immediately after surgery. The passive range of motion of the knee was initiated 2 to 6 weeks after the operation depending on X-ray images and patient compliance. Aggressive rehabilitation of the knee’s range of motion was usually begun 6 weeks after the operation and ended when the patient could bend the knee more than 90°.

### Data collection

Demographic data—including age, sex, involved side, and clinical outcomes—were obtained, and fracture comminution was recorded after consulting the operation findings and charts. The vertical fragment length and the preoperative fracture displacement were determined by consulting radiographs. Patient images were obtained and image features were measured using Digital Imaging and Communication in Medicine image-viewing software (πViewTM, INFINITT Co., Ltd., Seoul, South Korea). To prevent observation bias, two authors independently identified patients eligible for inclusion in each group and evaluated the outcomes independently. Disagreements were resolved through discussion with a third author.

The primary outcome of the study was radiologic loss of reduction, nonunion, implant removal, and clinical failure. The definition of radiological loss of reduction differed slightly between the two groups. In the TBW group, loss of reduction was defined as fracture displacement of more than 3 mm on a radiograph. However, for the TOR technique, the failure mechanism included not only fragment displacement but also recurrent avulsion of the patellar tendon. Thus, in the TOR group, loss of reduction was defined as (1) fracture displacement of more than 3 mm on a radiograph when compared with the postoperative image and (2) an IS ratio of > 1.2 during the follow-up period. The absence of a bridging callus over the fracture site at 6 months after the operation was recorded as nonunion. Clinical failure was noted if there was (1) loss of reduction that required revision osteosynthesis or (2) loss of reduction that resulted in permanent loss of the extensor mechanism.

The secondary outcome of the study was a change in the patellar height in patients without loss of reduction. After excluding patients with postoperative loss of reduction, we determined the IS ratio at 1 day, 3 months, and 6 months after surgery. Patella alta was defined as an IS ratio of > 1.2, whereas patella baja was defined as an IS ratio of < 0.8 [19].

Finally, we evaluated the risk factors for radiological loss of reduction after open reduction and internal fixation of inferior pole patellar fracture. The patients were assigned to subgroups with and without postoperative loss of reduction. The demographic data, fragment size, distance of fracture displacement, and fixation method of these subgroups were compared. Multivariable logistic regression was performed to identify the risk factors contributing to postoperative loss of reduction.

### Statistical analysis

Results were obtained using SPSS (SPSS Inc., USA). For the primary and secondary results, the chi-squared test was employed to evaluate differences in categorical variables, such as loss of reduction and nonunion. Continuous variables, including the size and displacement of fragments, were evaluated using the unpaired Student *t* test. The possible risk factors for radiological loss of reduction were identified, and cut-off points were determined by analyzing the receiver operating characteristic (ROC) curve. After this ROC analysis, the cut-off points in our model were age of 60 years, vertical length of fragment of 12 mm, displacement distance of 30 mm, female sex, and fracture comminution and fixation using the TOR technique. Odds ratios (ORs) were calculated to predict postoperative loss of reduction in a multivariable model. Results with *p* < 0.05 were considered statistically significant.

## Results

**Fig. 1:**
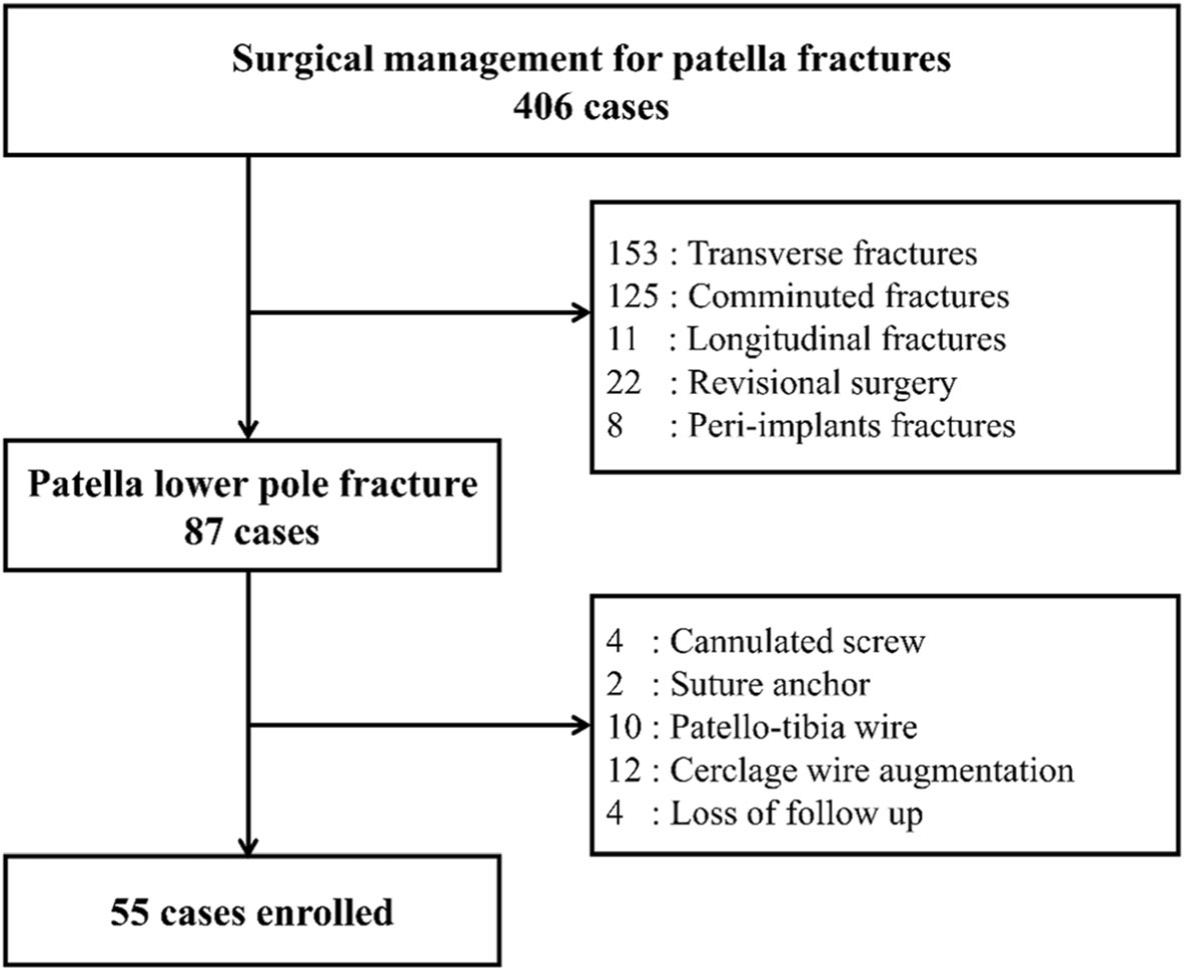
Patient recruitment flow chart

A total of 55 patients were enrolled, as shown in the flowchart presented in Fig. 1; of these patients, 30 were treated with TBW and 25 were treated with TOR. No significant differences were observed between the two groups in terms of age, sex, fracture comminution, preoperative displacement, or vertical fragment length (Table 1). In total, 11 patients (20%) developed radiological loss of reduction; among them, eight were in the TOR group. The mechanism of loss of reduction in the TOR group was fracture displacement in six patients and recurrent patellar tendon avulsion in two patients. Loss of reduction was significantly more prominent in the TOR group than the TBW group (*p* = 0.04). Nonunion occurred in 10 patients (18%), 7 of whom were in the TOR group (*p* = 0.09). In total, 18 patients in the TBW group (60%) requested removal of the implant due to irritation; no such request was made by any patient in the TOR group; this difference was significant (*p* < 0.001; Table 1). Two patients in the TBW group (7%) experienced clinical failure and underwent revision osteosynthesis. By contrast, three patients in the TOR group (12%) experienced clinical failure, but only one patient underwent revision osteosynthesis. The clinical failure rate did not differ significantly between the two groups (*p* = 0.49).

**Table 1:** Demographics data and postoperative complications of patients undergoing surgical management of inferior pole patellar fracture

Characteristic	TBW (*n* = 30)	TOR (*n* = 25)	*p*-value
Age	59.7 ± 14.1	55.3 ± 19.8	0.36
Gender (M/F)	10/20	13/12	0.16
Side (R/L)	13/17	13/12	0.36
Preoperative displaced (mm)	22.21, 15.71	27.24, 16.18	0.26
Vertical length of fragment (mm)	15.03, 2.80	13.78, 3.58	0.16
Fracture comminuted	11	12	0.40
Complications			
Loss of reduction	3	8	**0.04**
Nonunion	3	7	0.09
Removal of implants	18	0	**< 0.001**
Clinical failure	2	3	0.49

**Fig. 2:**
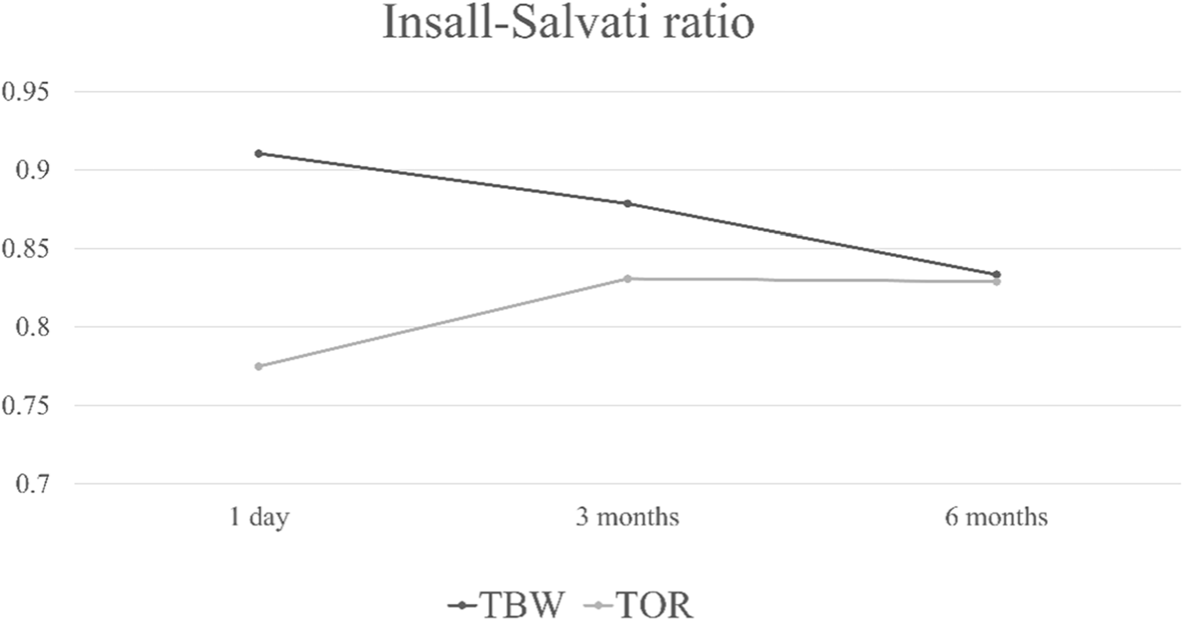
Insall–Salvati ratios 1 day, 3 months, and 6 months postoperation

On the first postoperative day, the IS ratio was significantly higher in the TBW group than in the TOR group (*p* = 0.01), and the average IS ratio in the TOR group indicated patella baja (0.77 ± 0.17). However, the IS ratio gradually decreased in the TBW group and increased in the TOR group (Table 2; Fig. 2). No significant differences were discovered 3 or 6 months after the operation.

**Table 2:** Insall–Salvati ratios 1 day, 3 months, and 6 months after surgery

Time	TBW (*n* = 27)	Transosseous reattachment (*n* = 17)	*p*-value
Post-op 1 day	0.91 ± 0.13	0.77 ± 0.17	**0.01**
Post-op 3 month	0.87 ± 0.12	0.83 ± 0.13	0.23
Post-op 6 month	0.83 ± 0.13	0.83 ± 0.17	0.92

We next compared the data of patients with and without radiological loss of reduction. No significant differences were observed in terms of age, sex, fracture comminution, or vertical fragment length. The preoperative displacement and vertical fragment length were significantly greater in the patients with loss of reduction than in those without (35.18 ± 14.23 mm vs. 21.83 ± 15.45 mm, *p* = 0.01). In addition, more patients in the loss of reduction group were treated using TOR than using TBW, and more patients without loss of reduction were treated using TBW than using TOR (*p* = 0.04; Table 3). Multivariable logistic regression was used to identify the ORs of the possible risk factors (Table 4). Fracture displacement of more than 30 mm was the only independent risk factor (OR 20.99, 95% confidence interval [CI] 3.01–146.40, *p* = 0.002). A fragment length of less than 12 mm (OR 9.43, 95% CI 0.89–100.06, *p* = 0.06) and fixation using TOR (OR 5.57, 95% CI 0.72–42.96, *p* = 0.10) also contributed to loss of reduction, but the associations were nonsignificant.

**Table 3:** Demographic data of patients with and without radiological loss of reduction

Characteristic	Loss of reduction (*n* = 11)	No loss of reduction (*n* = 44)	*p*-value
Age	61.0 ± 17.7	56.9 ± 16.8	0.48
Gender (M/F)	5/6	20/24	1.00
Fracture comminuted	5	18	0.79
Preoperative displaced (mm)	35.18 ±14.23	21.83 ±15.45	**0.01**
Vertical length of fragment (mm)	13.31± 3.46	14.75 ± 3.11	0.19
Surgical method			**0.04**
TBW	3	27	
Transosseous reattachment	8	17

**Table 4:** Multivariate logistic regression for postoperative radiological loss of reduction

Characteristic	Odd ratio (95% deviation)		*p*-value
Age > 60 years old	4.75	(0.60–37.67)	0.14
Female	1.17	(0.19–7.33 )	0.86
Displacement > 30 mm	20.99	(3.01–146.40)	**0.002**
Fragment length < 12 mm	9.43	(0.89–100.06)	0.06
Fracture comminution	1.03	(0.14–7.87)	0.98
Fixation with TOR	5.57	(0.72–42.96)	0.10

## Discussion

Surgery for fracture of the inferior pole of the patella is challenging because the fragments are typically small and comminuted, making reduction and fixation difficult. Although various surgical methods have been proposed, few studies have compared the results of TBW and TOR. This study compared the clinical and radiological results when these two techniques were used and identified the risk factors for postoperative loss of reduction.

Both TWB and TOR were discovered to be effective in treating fracture of the inferior pole of the patella, and they had a low clinical failure rate. However, TOR was significantly associated with radiological loss of reduction, and TWB was associated with implant removal. TOR with partial patellectomy has been widely used to treat inferior pole patellar fractures because the technique is simple and no special implants are necessary. However, TOR alone is thought to be insufficiently strong and additional augmentation should be applied. Massound et al. [13] reported complete fracture union after management with TOR plus circumferential wiring without partial patellectomy. However, breakage of the cerclage wire was observed in all patients, and the implants had to be removed due to irritation. Shrestha et al. [20] demonstrated that no major complications arose after inferior pole patellar fractures were managed with TOR using partial patellectomy and a patellotibial wire. Our results suggest that a favorable clinical result and low failure rate can be achieved using TOR alone without excision of the bony fragment even if the rate of radiological loss of reduction is higher than for TBW.

The TBW group exhibited low rates of nonunion and loss of reduction. Yang et al. [4] treated inferior pole patellar fracture by using TBW without partial patellectomy, but they employed a titanium cable with additional cable cerclage, which was tensioned to 60 N and locked. No nonunion, loss of reduction, or implant irritation was reported except for in eight patients who requested fixation removal due to personal reasons. Chang et al. [5] used anterior TBW through cannulated screws for the fixation of inferior pole patellar fracture and no loss of reduction or implant irritation was reported. The present results also suggest that TBW is an effective technique for treating inferior pole patellar fracture. However, more than half of patients may request implant removal.

**Fig. 3:**
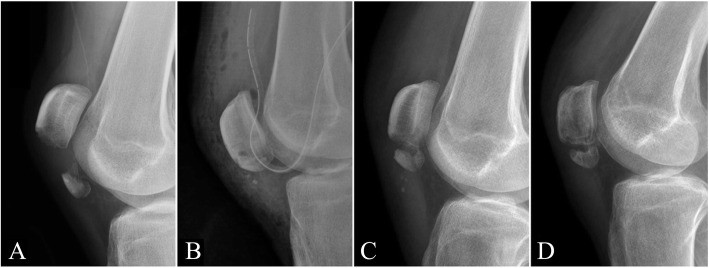
A man aged 33 years underwent transosseous reattachment for right patella inferior pole fracture. Knee radiographs **A** preoperation, **B** immediately after surgery, **C** 3 months postoperation, and **D** 9 months postoperation. Although postoperative radiological loss of reduction and nonunion were noted 3 months after surgery, patella height was maintained, and no further displacement was observed

Despite relatively high incidence of radiological loss of reduction in this study, the rate of clinical failure was low (4%). According to postoperative images, although radiological loss of reduction and nonunion developed in some cases, patellar height was adequate after more than 6 months of follow-up (Fig. 3). Pandey et al. [16] also reported good to excellent functional results after TOR with partial patellectomy followed by plaster protection for 4 to 5 weeks without radiological evaluation. Fibrotic healing may have been achieved because the patellar height was stable, rendering the joint sufficiently strong for general functional demands.

**Fig. 4:**
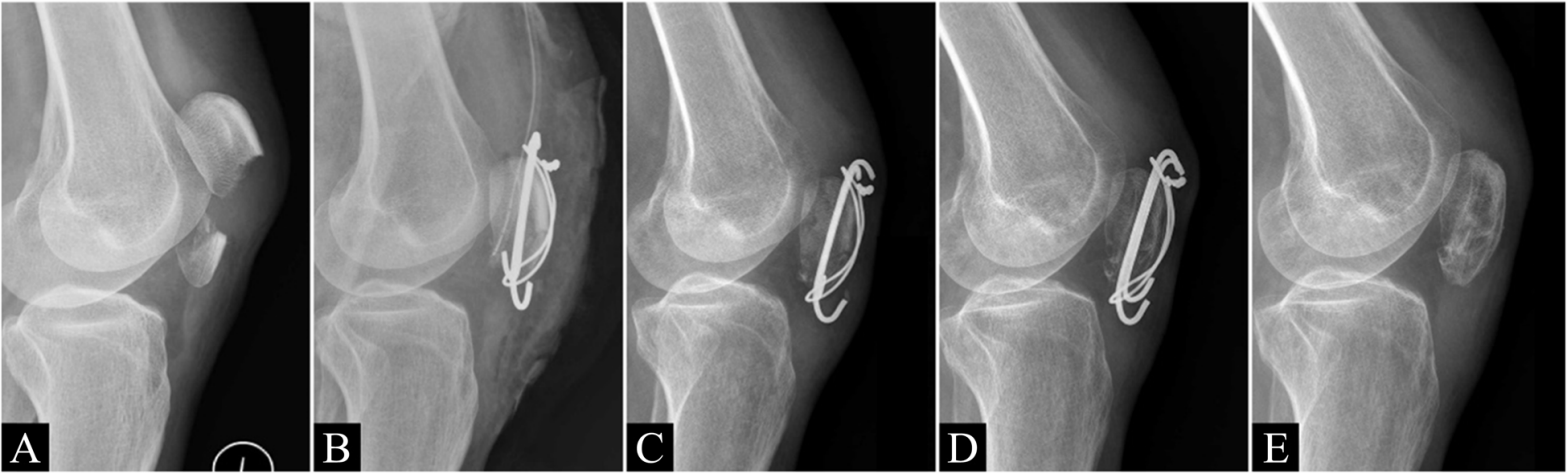
A women aged 53 years underwent tension band wire fixation for left patella lower pole fracture. Knee radiographs **A** preoperation, **B** immediately after surgery, **C** 3 months postoperation, **D** 12 months postoperation, and **E** after the removal of implants. The initial postoperative IS ratio was acceptable, but progressive patella baja was noted, even after the removal of implants

Patella baja has been reported to result from shortening of the patellar tendon fiber and traumatic and postoperative scarring [21, 22]. Moreover, patella baja was reported to be related to limited range of motion in extension and persistent anterior knee pain [23] and to be a complication after patellar fracture. Mariani et al. [22] identified postoperative patella baja in 12% of cases of patellar fracture. Lazaro et al. [17] noted patella baja in 57% of patients who underwent surgical treatment for patellar fracture. Kastelec et al. [10] revealed a significant decrease in the Blackburne–Peel ratio when inferior pole patellar fracture was treated with partial patellectomy, and patella baja was associated with unfavorable functional outcomes. However, in these studies, the researchers did not record the postoperative changes in patellar height. Therefore, we recorded the IS ratio 1 day, 3 months, and 6 months after the operation. Although patella baja was noted immediately after operation in the TOR group, the IS ratio increased over time. Moreover, the IS ratio decreased in the TBW group. This may have been because of irritation caused by the K-wire and wire around the tendon bone junction resulting in fibrotic change in the tendon fiber. Although the average IS ratio was in the normal range at 6 months after the operation, some patients in the TBW group developed patella baja even after the removal of implants (Fig. 4).

Finally, our results revealed that the only independent risk factor for radiological loss of reduction was fracture displacement of more than 30 mm. This result is reasonable because the displacement of the fragment is associated with the trauma energy and the severity of injury to soft tissue such as the patellar retinaculum and anterior joint capsule. Thus, prolonged immobilization may be necessary if the fracture displacement is more than 30 mm.

This study had some limitations. First, the data were collected retrospectively, and operations were performed by different surgeons. Individual surgeons may have had different preferences regarding the position of the K-wire or bone tunnel, and these differences may have slightly affected the outcomes [18]. The postoperative rehabilitation programs also differed slightly, possibly affecting the study outcomes, particularly fixation failure. We identified possible confounding variables, such as fragment length and distance of the fracture displacement, and we excluded patients with additional implants. However, related factors, such as bone quality and patient compliance, were not controlled for. Second, the sample was relatively small, and the results may lack sufficient power for the identification of meaningful differences. Third, the study mainly focused on radiologic results and simple reviews of medical records. Precise measurements of functional results such as range of motion and an injury-specific questionnaire should be applied in future research.

In conclusion, both TWB and TOR were effective in treating fracture of the inferior pole of the patella with a low clinical failure rate. Even TOR alone may increase the risk of radiological loss of reduction. By contrast, in 60% of patients undergoing TBW fixation, additional surgery was required to remove implants. Patella baja occurred immediately following TOR, but the IS ratio in the TOR group was similar to that in the TBW group after 3 months. Multivariable logistic regression revealed that fracture displacement of more than 30 mm was the only independent risk factor for postoperative radiological loss of reduction.

## Data Availability

The datasets used and/or analyzed during the current study are available from the corresponding author on reasonable request.

## Abbreviations

TBW: Tension band wire
TOR: Transosseous reattachment
IS: Insall–Salvati
K-wires: Kirschner wires
ROC: Receiver operating characteristic
ORs: Odds ratios
